# Probing the tunable multi-cone band structure in Bernal bilayer graphene

**DOI:** 10.1038/s41467-024-47342-0

**Published:** 2024-04-11

**Authors:** Anna M. Seiler, Nils Jacobsen, Martin Statz, Noelia Fernandez, Francesca Falorsi, Kenji Watanabe, Takashi Taniguchi, Zhiyu Dong, Leonid S. Levitov, R. Thomas Weitz

**Affiliations:** 1 1st Physical Institute, Faculty of Physics, University of Göttingen, Friedrich-Hund-Platz 1, Göttingen, Germany; 2 Research Center for Electronic and Optical Materials, National Institute for Materials Science, 1-1 Namiki, Tsukuba, Japan; 3 Research Center for Materials Nanoarchitectonics, National Institute for Materials Science, 1-1 Namiki, Tsukuba, Japan; 4 Department of Physics, Massachusetts Institute of Technology, Cambridge, Massachusetts, USA; 5 International Center for Advanced Studies of Energy Conversion (ICASEC), University of Göttingen, Göttingen, Germany

**Keywords:** Electronic properties and materials, Quantum Hall

## Abstract

Bernal bilayer graphene (BLG) offers a highly flexible platform for tuning the band structure, featuring two distinct regimes. One is a tunable band gap induced by large displacement fields. Another is a gapless metallic band occurring at low fields, featuring rich fine structure consisting of four linearly dispersing Dirac cones and van Hove singularities. Even though BLG has been extensively studied experimentally, the evidence of this band structure is still elusive, likely due to insufficient energy resolution. Here, we use Landau levels as markers of the energy dispersion and analyze the Landau level spectrum in a regime where the cyclotron orbits of electrons or holes in momentum space are small enough to resolve the distinct mini Dirac cones. We identify the presence of four Dirac cones and map out topological transitions induced by displacement field. By clarifying the low-energy properties of BLG bands, these findings provide a valuable addition to the toolkit for graphene electronics.

## Abstract (web summary)

Bernal-stacked bilayer graphene (BLG) has been extensively studied due to its tunable band gap and emerging electronic properties, but its low-energy band structure remains debated. Here, the authors report magnetotransport measurements of Bernal BLG, showing evidence of four Dirac cones and electrically induced topological transitions.

## Introduction

Graphene, a single layer of carbon atoms arranged in a hexagonal lattice, exhibits intriguing electronic properties due to its linearly dispersing bands forming Dirac cones at the K and K’ points. Yet, one of the key limitations of monolayer graphene is its zero bandgap, which renders it nonideal for digital electronic applications and controlling electronic interactions^1^. Several attempts have been made to artificially open up a bandgap in monolayer graphene, including chemical doping^2–4^, strain engineering^5–8^ and the creation of moiré patterns^9,10^. However, while these methods may allow to create a gap in an otherwise gapless dispersion, they also create disorder in the system or necessitate a complex experimental setup^10,11^. Opening a tunable band gap in pristine monolayer graphene by electrostatic gating presently appears to be out of reach since it would require electric field control with atomic precision to induce a potential difference between the two sublattices.

The simple Bernal-stacked bilayer graphene (BLG), to the contrary, does allow electrostatic tunability of a band gap and the high-energy parabolic dispersion – as shown by experimental spectroscopy and transport measurements^12–16^ as well as theoretical calculations^17,18^. However, perhaps surprisingly, there is no consensus between experiment and theory regarding the low-energy band structure of BLG. For example, quantum Hall measurements identified an eightfold degeneracy of the lowest Landau level (LLL), facilitated by a two-fold spin, valley and orbital degeneracy, consistent with a low-energy parabolic dispersion^15,19–22^. While such quantum Hall measurements provide some information about band symmetries, they leave several key questions unanswered. First, upon including higher-order hopping terms, at zero magnetic field and low carrier density one expects a metallic band that remains gapless at finite and not-too-strong displacement fields. This metallic fine-structure band features four Dirac cones with different chiralities at each valley and prominent van Hove singularities^19,23–26^, which appear inconsistent with the picture inferred from quantum oscillations. Furthermore, there are outstanding questions about strong exchange-driven phases in suspended bilayer graphene^15,20,22,27,28^ which are hard to reconcile with linear dispersion at low energies. Lastly, signatures of changes in Fermi surface topology due to trigonal warping have been identified in the case of strong displacement fields where sizable bandgaps are opened. Specifically, an unusual ordering of quantum Hall states is observed in the presence of a strong electric displacement field in strongly biased BLG^29–32^, Bernal trilayer graphene^33–35^, rhombohedral trilayer graphene^36,37^ and Bernal tetralayer graphene^38^, which is consistent with the theoretical band structure predicting a trigonally-warped low-energy Fermi surface topology.

The present work aims to resolve the puzzle of why the linearly dispersing bands and electric-field-induced changes of topology have not been observed experimentally in BLG. Signs of topological changes in the band structure involving a van Hove singularity should be detectable, e.g., in a quantizing magnetic field in which the presence of four Dirac cones results in an exotic sequence of Landau levels distinct from the previously studied instances of Landau levels. Accessing this complex field-tunable band structure with multiple Dirac cones and field-tunable van Hove singularities is of clear interest for the physics of strongly correlated systems as well as graphene band engineering.

## Results

To reveal the detailed low-energy band structure and detect electric-field controllable linear dispersing bands, here we have chosen to work with the hexagonal boron nitride (hBN) encapsulated Bernal-stacked BLG sample system over suspended BLG—even though both systems yield samples of similar quality. In the suspended BLG, however, due to the low-dielectric constant of the dielectric (vacuum), the exchange energy scale seems to dominate the low-energy physics leading to a variety of nontrivial groundstates^15,20,22,27,28^. Our encapsulated BLG is equipped with graphite top and bottom gates and two-terminal graphite contacts (see “Methods” and Supplementary Fig. 1 and 2). All measurements were performed in a cryostat at a temperature of 10 mK employing standard lock-in techniques at 78 Hz and an ac bias current of 1 nA. By varying both gate voltages, we were able to tune the charge carrier density (*n*) and the electric displacement field (*D*) independently. Since we, on the one hand, use two-terminal measurements (i.e., contact resistance cannot be easily determined) and, on the other hand, explore the behavior at relatively low *B* and down to *B* = 0, the respective LL are not fully developed (quantized), we have found it convenient to use d*G*/d*n* values as the markers of the band structure. We complement our measurements with tight binding band structure calculations of the quantum Hall states in BLG expected at zero and low *D*-fields. We use a standard BLG bandstructure model in which we include also the weaker inter-layer coupling parameters *γ*_*3*_ and *γ*_*4*_ as well as an energy difference between dimer and non-dimer atomic sites Δ^17,39^ (see “Methods” for technical details of the calculations).

### Tight binding calculations of Landau Levels

**Fig. 1:**
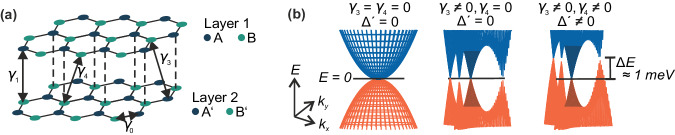
Lattice and band structure of Bernal bilayer graphene. **a** Lattice structure of Bernal bilayer graphene. The interlayer hopping processes described by the parameters ${\gamma }_{0},{\gamma }_{1},{\gamma }_{3}$ and ${\gamma }_{4}$ are indicated. **b** Band structure of bilayer graphene in an energy (*E*) range of −2 mV to +2 mV at zero electric displacement field (*D* = 0) calculated with a tight binding model including various subsets of hopping parameters and on-site parameter Δ’, featuring four Dirac cones of different chiralities and three van Hove singularities in each valley. The center cones are shaded darker.

**Fig. 2:**
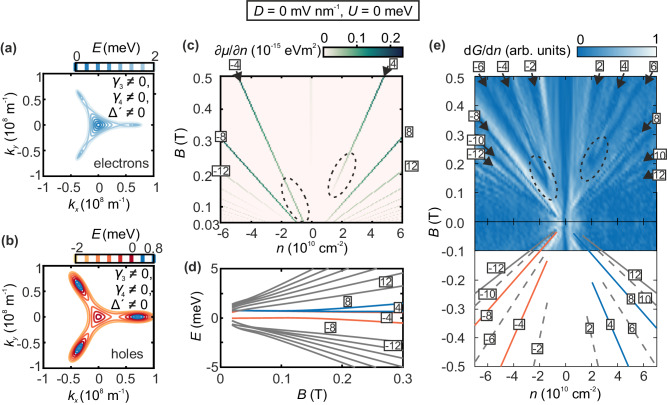
Fermi surface contours and quantum Hall states of Bernal bilayer graphene at *D* = 0. **a, b** Fermi surface contour of the conduction band (**a**) and valence band (**b**) of bilayer graphene at different Fermi energy levels. All hopping parameters (${\gamma }_{0},{\gamma }_{1},{\gamma }_{3}$ and ${\gamma }_{4}$) and the on-site parameter Δ’ are included. **c** Calculated inverse compressibility (∂μ/∂n) as a function of charge carrier density and magnetic field at zero electric displacement field (*D* = 0) and temperature *T* = 0.1 K. The corresponding quantum Hall states are labeled by numerals. The regions in which quantum Hall states with filling factors ν = ±4 terminate are highlighted by dashed circles. **d** Evolution of Landau levels as a function of magnetic field at *D* = 0. The four lowest Landau levels are colored, whereas Landau levels contributing to hole transport are colored in red and Landau levels contributing to electron transport are colored in blue. The lowest red colored Landau level originates from the center mini Dirac cone, the other three lowest Landau levels originate from the three off-center mini Dirac cones. Filling factors are indicated by numerals. A larger version of this plot is shown in Supplementary Fig. 3. **e** Derivative of the normalized conductance measured as a function of the charge carrier density and the magnetic field at *D* = 0. Blue regions correspond to vanishing differential conductance (i.e., a conductance plateau). The slopes of the lowest quantum Hall states are traced by lines in the mirror image. Solid lines correspond to zero-interaction (free-particle) quantum Hall states that allow for comparison with theory (Fig. 2c). Their colors are adapted from Fig. 2d. Dashed lines correspond to interaction-induced quantum Hall states in which the spin or valley degrees of freedom is broken. The regions in which quantum Hall states with filling factors ν = ±4 end are highlighted by dashed circles.

We first discuss the tight binding calculations of LLs that are used below to experimentally identify the transition from a metallic band with four Dirac cones to a gapped parabolic dispersion. In the absence of an interlayer potential difference *U* and in case trigonal warping effects are ignored or are made irrelevant by disorder, the low-energy band structure of BLG exhibits a nearly parabolic dispersion (Fig. 1b (left))^15,17,23^. Consistent with previous experiments conducted in quantizing magnetic fields *B* > 0.5 T^15,19,27^, this leads to an eight-fold degeneracy of the lowest Landau level (LLL) due to spin, valley and orbital degrees of freedom, and to a four-fold degeneracy of all higher Landau levels due to spin and valley degrees of freedom (see “Methods”). In case that the low-energy band structure at the charge neutrality point can be resolved below a Fermi energy *E* ~ ±1 meV, the band structure dramatically changes when including trigonal warping, and three off-center and one center cones emerge in each valley (also referred to as mini Dirac cones), resulting from the weaker skew interlayer hopping term *γ*_*3*_. The four cones with a Dirac-like spectrum resemble a four-fold copy of the spectrum of monolayer graphene, for which the LLL is shared equally by electrons and holes, overall leading to a 16-fold degeneracy (2 spins, 2 valleys, 4 mini Dirac cones). This would result in the appearance of quantum Hall states with filling factors $\nu$ = $\frac{{{{{{\rm{h}}}}}}n}{{{{{{\rm{e}}}}}}B}$ = ±8^19,23–25^. In addition, the skew interlayer hopping term *γ*_*4*_ and the on-site parameter Δ’, describing the energy difference between atoms A and A’ or B and B’, create an energetic asymmetry between these cones^17^. While the center cone formed by the conduction and valence bands occurs at zero energy, the off-center cones occur at higher energies (Fig. 1b, Fig. 2a, b; more information on the impact of *γ*_*3*_*, γ*_*4*_, and Δ’ is given in Supplementary Fig. 3). In quantum Hall measurements, these changes in the band structure can be discerned only at *B* < 0.2 T since here, the inverse of the magnetic length ${l}_{B}=\sqrt{\hslash /({eB})}$ with $\hslash=h/2\pi$ and Planck’s constant *h* is smaller than the distance in momentum space between two adjacent mini Dirac cones (i.e., below the fields at which magnetic breakdown occurs)^31,32^.

Figure 2c shows the calculated inverse compressibility (∂*μ*/∂*n* with chemical potential *μ, n* charge carrier density) as function of *n* and *B* at *D* = 0 (Landau fan diagram). Here, larger energy gaps in the Landau level spectrum (Fig. 2d) manifest as prominent peaks corresponding to quantum Hall states that are labeled by numerals (the calculations include *γ*_*0*_, *γ*_*1*_, *γ*_*3*_, *γ*_*4*_ and Δ’, spin splitting is included manually in the LL for the figures, both valleys are included in the calculation and are fully degenerate; see “Methods” for further details). While quantum Hall states with $\nu$ = ±8 indeed exhibit the largest compressibility and go down to the lowest *B* in the valence/ conduction band also the quantum Hall state with $\nu$ = −4 (but not $\nu$ = +4) is very robust, i.e., it can be resolved until very low *B* (Fig. 2c), which is a manifestation of the electron-hole asymmetry. Neglecting the spin and valley degrees of freedom, the three off-center cones exhibit a three-fold degenerate LLL and are shifted to higher energies. Thus, the center cone LLL is non-degenerate with the LLL that belong to the off-center cones (Fig. 2d). Since the LLL is shared between electrons and holes, the non-degenerate center cone as well as one of three LL originating from the three off-center cones contribute to hole transport and give rise to quantum Hall states with $\nu$ = −8 and $\nu$ = −4 respectively. The other two LL stemming from the three off-center cones contribute to electron transport and give rise to a quantum Hall state with $\nu$ = +8. The quantum Hall state with $\nu$ = +4 only emerges at larger *B* where the degenerate LL diverge. With increasing *n*, the conventional sequence quantum Hall states with filling factors $\nu$ = ±12, ±16, ±20 is recovered and the Fermi level lies above the Lifshitz transition where the Fermi surface is fully connected.

### Quantum Hall measurements at zero displacement field

The theory thus shows in detail how the presence of four Dirac cones can unambiguously be identified in experiment. Figure 2e shows the normalized derivative of the measured two-terminal conductance |dG/dn| as a function of *n* and *B* at *D* = 0 V/nm. Quantum Hall states appear as plateaus in the conductance and thus as dips in |dG/dn| and can be assigned by their corresponding slopes in the Landau fan diagram (see “Methods”). Consistent with our theoretical simulations, quantum Hall states with $\nu$ = ±8 are the most robust and can be observed at the smallest *B*, down to *B* ≈ 0.05 T which reveals the presence of four mini Dirac cones. Additionally, due to electron-hole asymmetry, the quantum Hall state with $\nu$ = −4 appears at slightly larger magnetic fields (B ≈ 0.15 T), while the $\nu$ = +4 quantum Hall state only appears above 0.2 T when the magnetic breakdown occurs (indicated by dashed lines in Fig. 2c, e). At magnetic fields above 0.3 T, a sequence of even integer quantum Hall states appears which is consistent with previous measurements in freestanding BLG^15,20,27,40^ and which reveals the high quality of our sample. Here, the spin degeneracy is likely lifted due to Coulomb interactions resulting in a two-fold degeneracy (valley) instead of the predicted four-fold degeneracy (spin and valley)^23,41,42^. Notably, some of the non-four-fold degenerate quantum Hall states including the quantum Hall states with ν = ±6 also go down to below 0.3 T and then merge with the quantum Hall states with ν = ±4 and demand further investigation. Since spin and valley splitting are both neglected in our theoretical simulations, this two-fold degeneracy is only observed in our experimental data but not visible in the calculated inverse compressibility.

### Landau level spectrum at finite displacement field

**Fig. 3:**
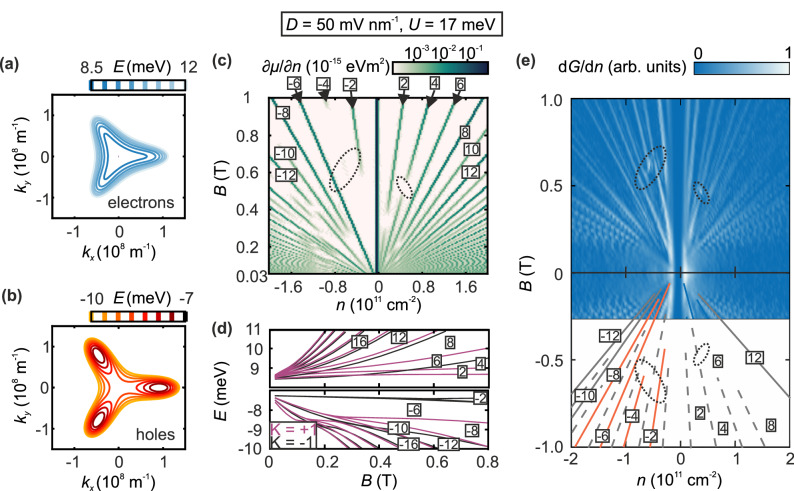
Band structure and quantum Hall states of bilayer graphene at finite *D.* **a, b** Fermi surface contour of the conduction band (**a**) and valence band (**b**) of bilayer graphene at different Fermi energy levels at *U* = 17 meV. **c** Calculated inverse compressibility (∂μ/∂n) as a function of charge carrier density and magnetic field at *U* = 17 meV and temperature *T* = 0.1 K. The corresponding quantum Hall states are labeled by numerals. Regions corresponding to Landau level crossings are marked by dotted circles. **d** Evolution of Landau levels as a function of the magnetic field. Landau levels contributing to transport in valley K = 1 are colored in purple, Landau levels contributing to transport in valley K = −1 are colored in grey. **e** Derivative of the normalized conductance measured as a function of the charge carrier density and the magnetic field at *D* = 50 mV/nm. Solid lines correspond to the non-interaction induced quantum Hall states that allow for comparison with theory (Fig. 3c). Dashed lines correspond to interaction-induced quantum Hall states in which the spin degree of freedom is broken. The corresponding quantum Hall states are labeled by numerals. The regions corresponding to Landau level crossings are marked by dotted circles.

While the measurements at zero displacement fields show the existence of four Dirac cones near charge neutrality, we now show the tunability of the Dirac cones followed by bandgap opening controlled by *D*. We note in passing that for very small D-fields while the four Dirac cones will be individually gapped, there will be no overall gap in the spectrum due to the inherent energetic offset between the center and the three off-center Dirac cones. While theoretical tight binding calculations of this regime are discussed in the Methods and are shown in Supplementary Fig. 4, this regime is not within our experimental resolution. We first discuss our calculations and corresponding charge transport measurements at constant small *D*, where already an overall bandgap has opened up in the previously linear Dirac spectrum of BLG. This goes along with drastic changes of the band structure: the center cone diminishes whereas the three off-center cones (Fig. 3a, b) with however a parabolic dispersion remain, which we consequently refer to as pockets.

In the valence band, where the three off-center cones already dominate at *D* = 0 due to electron-hole asymmetry, the number of pockets changes from four to three at finite *D* (Fig. 3b), resulting in a reordering of expected quantum Hall states (Fig. 3c–e)^39,43^. We expect the LLL to be six-fold degenerate at low *B* due to the remaining three leg pockets (3 pockets, 2 spins, the 2 valleys are degenerate, Fig. 3d, e)^31,32^ and the $\nu$ = −6 quantum Hall state is expected to be the most robust for hole doping (Fig. 3c). These theoretical considerations are confirmed by our measurements. As shown in Fig. 3e, for *D* = 50 mV/nm, the $\nu$ = −6 quantum Hall state can be resolved down to very low magnetic fields of *B* = 100 mT. Surprisingly, this also holds for the $\nu$ = −3 quantum Hall state which could result from spin or valley polarization at low *B* and low *n* due to Stoner ferromagnetism that can occur in the vicinity of the Lifshitz transition^29,30,44^. At *B* = 600 mT, a sudden change in the degeneracy of Landau level takes place for *n* < 0, which can be attributed to the magnetic breakdown. Here the effects of the trigonal warping are no longer relevant, and we can observe all integer quantum Hall states. It is worth noting that at larger densities, quantum Hall states with $\nu$ = −7 and $\nu$ = −9 appear below *B* = 500 mT. In this regime, the Fermi energy level lies above the Lifshitz transition and quantum Hall states start to become valley and spin polarized with increasing magnetic field.

The effects of band flattening and disappearing of the low-energy Dirac spectrum can be also seen in the conduction band (Fig. 3a) where the center pocket also becomes less prominent with increasing *D*. However, due to electron-hole asymmetry the center cone is still dominating at *U* = 17 meV and the band becomes flatter with increasing *U* until *U* ≈ 60 meV^45^. At *U* = 17 meV, the degeneracy of quantum Hall states is not as much affected by trigonal warping as in the valence band and quantum Hall states with even ν appear first in the magnetic field in our conductance measurements (Fig. 3e). Remarkably, the quantum Hall states with ν = +3 and ν = +4 disappear at a magnetic field of 0.5 T and then reemerge at about 0.6 T (Fig. 3c, e) resulting from a crossing of two bands that correspond to different valleys (Fig. 3d).

### Controlling the *D*-field induced Lifshitz transitions

**Fig. 4:**
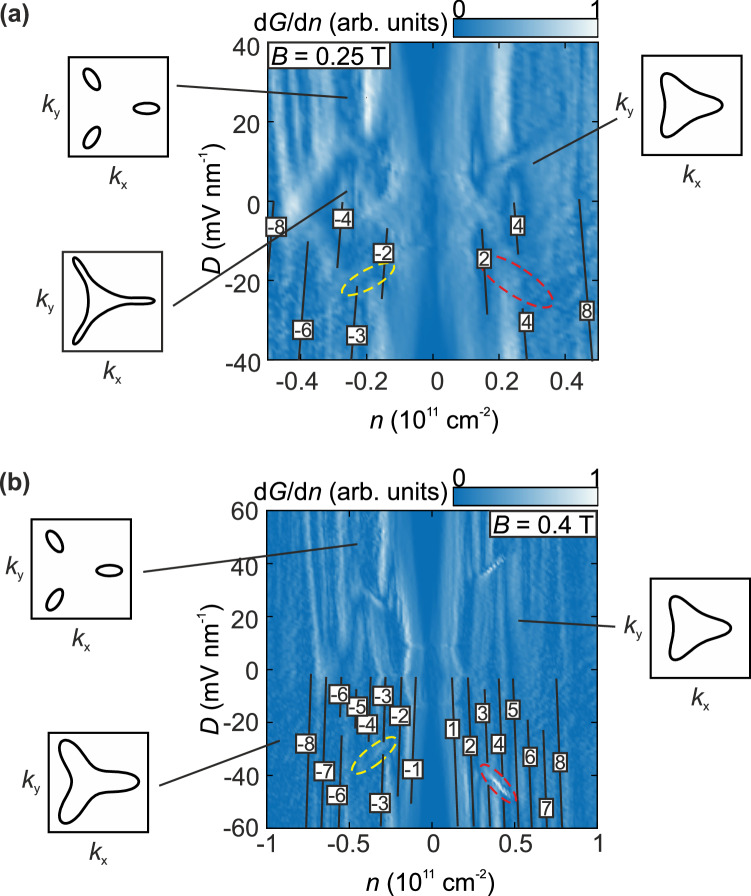
Changing the degeneracy of quantum Hall states due to magnetic breakdown induced by an electric displacement field. **a, b** Derivative of the normalized conductance measured as a function of the charge carrier density and electric displacement field at *B* = 0.25 T (**a**) and *B* = 0.4 T (**b**). Quantum Hall states are labeled by numerals and traced by lines. Note, that the quantum Hall states are symmetric for positive and negative values of *D* but the labelling was restricted to *D* < 0 for better visibility. Transitions between quantum Hall states due to trigonal warping and electron-hole asymmetry are highlighted by yellow dotted lines, crossings between Landau levels of different K valleys are highlighted by red dotted circles. Quantum Hall states are three-fold degenerate at large *D* where the pockets are more pronounced, and the magnetic breakdown has not yet accord. Schematics of Fermi contours corresponding to regions which different Fermi surface topologies are shown in the insets. Apart from the number of Fermi surfaces, the degeneracy of quantum Hall states is also affected by spin and valley polarization at large *B* and *D*.

The active control and lifting of the four-fold Dirac spectrum can be also traced by controlling the displacement field at constant B (Fig. 4a, b). For example, at *B* = 0.25 T (Fig. 4a) the magnetic breakdown has already occurred for low *D* resulting in the appearance of eight-fold degenerate quantum Hall states at *D* = 0 mV/nm (2 valleys, 2 spins, 2 orbits). At 3 mV/nm <|D | < 25 mV/nm the quantum Hall state with ν = ±2 appears due to valley polarization^27^ while at |*D* | > 20 mV/nm the three pockets can be resolved individually and a crossover from a two-fold (two spins) to a three-fold degenerate Landau level spectrum (three pockets) appears at hole doping (yellow circle in Fig. 4a). Higher LL are four-fold degenerate (2 valleys, 2 spins) above a Lifshitz transition since their corresponding Fermi surface is fully connected. For larger *B*, e.g., at *B* = 0.4 T (Fig. 4b), the crossover to the parabolic shifts to larger *D* where the three pockets are more pronounced. Here also the crossings of LL stemming from different valleys in the conduction band can be discerned in the *n* vs. *B* plot.

## Discussion

In conclusion, we present measurements demonstrating that bilayer graphene exhibits a highly tunable band structure at low energies where four distinct Dirac cones, under tuning transverse field, undergo topological transitions and merge into a parabolic band or three pockets with a gapped parabolic dispersion. The topological transitions result in a complex series of Landau levels that we extract by virtue of numerical diagonalization methods based on a realistic tight binding model and measurements in high-quality hBN-encapsulated samples. These results show that the simple and seemingly well-understood Bernal bilayer graphene is a true example of a long-sought tunable Dirac material with linear dispersion at low energies and an accessible band gap of up to 250 meV^14^. This, coupled with the presence of tunable van Hove singularities and a cascade of correlated states emerging at large *D* (that have been extensively discussed in earlier works^29,30,44,45^), makes BLG a promising material for exploring ordered states of interacting electrons. Furthermore, these attributes make BLG an attractive material for developing low-energy fast electronics, including graphene-based digital logic devices with true off-states^1,46^ and improved types of graphene transistors in which the mobility characteristics of monolayer graphene remain uncompromised^1^. The role of electron interactions and trigonal warping effects, in particular their impact on the renormalized many-body band structure, is an interesting topic for future work that can be addressed in high-quality freestanding BLG samples where interaction effects dominate even at low electric fields^15,27^.

## Methods

### Tight-binding calculations

The Landau level calculations were based on a realistic tight binding model for the $\pi$-electrons as described in ref. ^17^.

The model includes different hopping processes which are described by different parameters: ${\gamma }_{0}$ describes the tunneling for neighboring sites within a single graphene sheet; ${\gamma }_{1}$ accounts for hopping processes between the aligned lattice sites of the two sheets, the so-called dimer sites. Finally, ${\gamma }_{3}$ and ${\gamma }_{4}$ are the hopping parameters between non-dimer sites; ${\gamma }_{3}$ causes the trigonal warping of the Fermi sea at low carrier densities. Further parameters are ${\Delta }^{{\prime} }$, an energy difference between dimer and non-dimer sites, and the interlayer potential $U$ accounting for an out-of-plane displacement field. The magnitudes of these parameters were taken from ref. ^39^.

We used a two-band model which is reduced to the non-dimer sites and includes direct hoppings via ${\gamma }_{3}$ and ${\gamma }_{4}$, as well as hoppings via dimer sites. It is expected to capture the correct low-energy physics if ${\gamma }_{0}$ and ${\gamma }_{1}$ are the relevant energy scales of the problems.

Writing $\pi=\xi {p}_{x}+i{p}_{y}$ and ${\pi }^{+}=\xi {p}_{x}-i{p}_{y}$, where *ξ* *=* +1,−1 denotes the valley index and ${p}_{x}$ and ${p}_{y}$ are the x and y components of the momentum vector, one may decompose the Hamiltonian as $h={h}_{0}+{h}_{w}+{h}_{{as}}+{h}_{U}$ with

1 $${h}_{0}=-\frac{1}{2m}\left(\begin{array}{cc}0 & {\left({\pi }^{+}\right)}^{2}\\ {\pi }^{2} & 0\end{array}\right)$$

2 $${h}_{w}={v}_{3}\left(\begin{array}{cc}0 & \pi \\ {\pi }^{+} & 0\end{array}\right)-\frac{{v}_{3}a}{4\sqrt{3}{{\hslash }}}\left(\begin{array}{cc}0 & {\left({\pi }^{+}\right)}^{2}\\ {\pi }^{2} & 0\end{array}\right)$$

3 $${h}_{{as}}=\left(\frac{2v{v}_{4}}{{\gamma }_{1}}+\frac{{\Delta }^{{\prime} }{v}^{2}}{{\gamma }_{1}^{2}}\right)\left(\begin{array}{cc}{\pi }^{+}\pi & 0\\ 0 & \pi {\pi }^{+}\end{array}\right)$$

4 $${h}_{U}=-\frac{U}{2}\left[\left(\begin{array}{cc}1 & 0\\ 0 & -1\end{array}\right)-\frac{2{v}^{2}}{{\gamma }_{1}^{2}}\left(\begin{array}{cc}{\pi }^{+}\pi & 0\\ 0 & -\pi {\pi }^{+}\end{array}\right)\right]$$

where we have introduced the velocities $v=\frac{\sqrt{3}a{\gamma }_{0}}{2\hslash }$, ${v}_{3}=\frac{\sqrt{3}a{\gamma }_{3}}{2\hslash }$, ${v}_{4}=\frac{\sqrt{3}a{\gamma }_{4}}{2\hslash }$ with the lattice constant $a$ and the band mass parameter $m=\frac{{\gamma }_{1}}{2{v}^{2}}$. A diagonalization yields momentum space representations of two component wave functions **Ψ** = $\frac{1}{\sqrt{2}}\left({\psi }_{1},{\psi }_{2}\right)$, with the components ${\psi }_{1\left(2\right)}$ specifying the wave functions projections on the non-dimer sites.

The first term (${h}_{0}$) describes a simple parabolic two-band model with the dominant hopping processes between non-dimer sites, the second (${h}_{w}$) accounts for trigonal warping and adds a small correction to the parabolic term, the third one (${h}_{{as}}$) introduces an intrinsic electron-hole asymmetry and the fourth one (${h}_{U}$) describes coupling to external fields.

The effect of the latter may be understood by realizing that a displacement field results in a potential difference $U$ between the two graphene sheets. A rigorous estimation of the magnitude of the potential requires a self-consistent computation that includes the screening effects due to the redistribution of charge carriers between the two sheets in the presence of a displacement field^17^.

Without aiming for an exact quantitative description, we estimate the rough magnitude of $U$ via a simple plate-capacitor calculation as *U* *=* *ecD* where *c* *=* 3.35 Å is the interlayer spacing. This estimate assumes minor importance of screening effects at small displacement fields.

The out-of-plane magnetic field of magnitude $B$ is introduced by adding a contribution from the vector potential to canonical momenta. In the Landau gauge this modifies the momentum operator according to $\pi=-i\xi \hslash {\partial }_{x}+\hslash {\partial }_{y}-{ieBx}$ and ${\pi }^{+}=-i\xi \hslash {\partial }_{x}-\hslash {\partial }_{y}+{ieBx}$. The operators satisfy the same algebraic relation as the ladder operators of the harmonic oscillator, namely [π,π^+^] *=* −*2ℏBeξ*. One may use this fact to define the operators $a=-\frac{i}{\sqrt{2\hslash {eB}}}\pi$ and ${a}^{+}=\frac{i}{\sqrt{2\hslash {eB}}}{\pi }^{+}$ for the ${K}_{+}$-valley or ${a}^{+}=-\frac{i}{\sqrt{2\hslash {eB}}}\pi$ and $a=-\frac{i}{\sqrt{2\hslash {eB}}}{\pi }^{+}$ for the ${K}_{-}$-valley. These operators act like raising and lowering operators for an oscillator with the cyclotron frequency ${\omega }_{c}=\frac{{eB}}{m}$. Their action on the oscillator wave functions reads $a{{{{{\phi }}}}}_{{{{{n}}}}}=\sqrt{n}{{{{{\phi }}}}}_{{{{{n}}}}{{{{-}}}}{{{{1}}}}}$ and ${a}^{+}{{{{{\phi }}}}}_{{{{{n}}}}}=\sqrt{n+1}{{{{{\phi }}}}}_{{{{{n}}}}{{{{+}}}}{{1}}}$

The Hamiltonian in the ${K}_{+}$-valley may then be rewritten as

5 $$\hat{h}=\left[\begin{array}{cc}-\frac{U}{2}+{C}_{+}{a}^{+}a & A{({a}^{+})}^{2}-{iRa}\\ A{a}^{2}+{iR}{a}^{+} & \frac{U}{2}+{C}_{-}a{a}^{+}\end{array}\right]$$

with

6 $$A=\hslash {\omega }_{c}\left(1+\frac{{\gamma }_{1}{\gamma }_{3}}{6{\gamma }_{0}^{2}}\right)$$

7 $$R=\frac{{\gamma }_{3}}{{\gamma }_{0}}\sqrt{{\gamma }_{1}\hslash {\omega }_{c}}$$

and

8 $${C}_{\pm }=\hslash {\omega }_{c}\left(\frac{2{\gamma }_{4}}{{\gamma }_{0}}+\frac{{\Delta }^{{\prime} }\pm U}{{\gamma }_{1}}\right)$$

In the opposite valley, the roles of creators and annihilators are interchanged.

In the presence of trigonal warping interaction, the eigenvalue problem has no analytic solution. In order to get a numerical solution, we used a matrix representation of the Hamiltonian in a truncated basis. This method is expected to give good results for the low-energy spectrum, as the discarded high-energy states do not hybridize with those at low energy.

In the K_+_-valley, using

9 $$\left\{\left|0\bigg\rangle=\left[\begin{array}{c}{\phi }_{0}\\ 0\end{array}\right],\right|1\bigg\rangle=\left[\begin{array}{c}{\phi }_{1}\\ 0\end{array}\right],\left|n,{{{{{\rm{\sigma }}}}}}\bigg\rangle=\frac{1}{\sqrt{2}}\left[\begin{array}{c}{\phi }_{{{{{{\rm{n}}}}}}}\\ \sigma {\phi }_{{{{{{\rm{n}}}}}}-2}\end{array}\right]\right|(n\ge 2) \ ,\sigma=\pm \right\}$$

the matrix elements evaluate to

10 $$\left\langle 0\left|h\right|0\right\rangle=\frac{-U}{2}\ \ \left\langle 3 ,\sigma \left|h\right|0\right\rangle=\frac{\sigma {iR}}{\sqrt{2}}$$

11 $$\left\langle 1\left|h\right|1\right\rangle=\frac{-U}{2}+{C}_{+}\ \ \left\langle 4,\sigma \left|h\right|1\right\rangle=\sigma {iR}$$

12 $$\left\langle n,\sigma \left|h\right|n,{\sigma }^{{\prime} }\right\rangle=	 \sigma {\delta }_{\sigma {\sigma }^{{\prime} }}A\sqrt{n\left(n-1\right)}-\frac{U}{2}\left(1-{\delta }_{\sigma {\sigma }^{{\prime} }}\right) \\ 	+\frac{1}{2}\left({C}_{+}n+\sigma {\sigma }^{{\prime} }{C}_{-}\left(n-1\right)\right)$$

13 $$\left\langle n+3,\sigma \left|h\right|n,{\sigma }^{{\prime} }\right\rangle=\frac{\sigma {iR}\sqrt{n+1}}{2}$$

All remaining matrix elements follow the requirement of the matrix being Hermitian.

Analogously, for the ${{{{{{\rm{K}}}}}}}_{-}$-valley we used

14 $$\left\{\left|0\bigg\rangle=\left[\begin{array}{c}0\\ {\phi }_{0}\end{array}\right],\right|1\rangle=\left[\begin{array}{c}0\\ {\phi }_{1}\end{array}\right],\bigg|n,{{{{{\rm{\sigma }}}}}}\rangle=\frac{1}{\sqrt{2}}\left[\begin{array}{c}{\phi }_{{{{{{\rm{n}}}}}}-2}\\ {{{{{\rm{\sigma }}}}}}{\phi }_{{{{{{\rm{n}}}}}}}\end{array}\right]\bigg|(n\ge 2),\, \sigma=\pm \right\}$$

to obtain

15 $$\left\langle 0\left|h\right|0\right\rangle=\frac{U}{2}\ \ \left\langle 3,\sigma \left|h\right|0\right\rangle=\frac{-{iR}}{\sqrt{2}}$$

16 $$\left\langle 1\left|h\right|1\right\rangle=\frac{U}{2}+{C}_{-}\ \ \left\langle 4,\sigma \left|h\right|1\right\rangle=-{iR}$$

17 $$\left\langle n , \sigma \left|h\right|n,{\sigma }^{{\prime} }\right\rangle= 	 \, \sigma {\delta }_{\sigma {\sigma }^{{\prime} }}A\sqrt{n\left(n-1\right)}-\frac{U}{2}\left(1-{\delta }_{\sigma {\sigma }^{{\prime} }}\right) \\ 	 \ + \frac{1}{2}\left({C}_{+}\left(n-1\right) + \sigma {\sigma }^{{\prime} }{C}_{-}n\right)$$

and

18 $$\left\langle n+3 , \sigma \left|h\right|n,{\sigma }^{{\prime} }\right\rangle = \frac{-{\sigma }^{{\prime} }{iR}\sqrt{n+1}}{2}$$

For the calculation, an upper cutoff for the Landau level index was set at ${n}_{\max }=300$ by observing the convergence behavior of the low-lying energy levels.

To illustrate the impact of ${\gamma }_{3}$, ${\gamma }_{4}$, and ${\Delta }^{{\prime} }$ on the low-energy band and Landau levels, the band structure is shown in Supplementary Figs. 3 and 5 with ${\gamma }_{3}$, ${\gamma }_{4}$, and ${\Delta }^{{\prime} }$ not included in (a), with only ${\gamma }_{3}$ included in (b) and ${\gamma }_{3}$, ${\gamma }_{4}$, and ${\Delta }^{{\prime} }$ all included in (c). Also, Supplementary Figs. 3 and 5 show the evolution of Landau levels as a function of the magnetic field, as well as the inverse compressibility in the quantized Hall regime that was calculated as a function of charge carrier density and magnetic field.

The electron-hole asymmetry in the band structure in combination with trigonal warping gives rise to a semi-metallic behavior at a low interlayer bias *U*. The interlayer bias gaps out the cones individually, however if sufficiently small, the gaps do not exceed the energetic offset of the leg pocket with respect to the center pocket, such that a global gap only emerges at a certain threshold value of U ≈ 1 meV (Supplementary Fig. 4). Below this interlayer potential, a finite density of states remains in the overlap region of the electron and the hole band. Owing to the small energy window for this phenomenon to appear, this regime is not directly accessible by spectroscopy methods. The magnetotransport measurements presented in the main text supported by the Landau level calculations serve as an indirect proof for the existence of this regime.

### Calculation of the inverse compressibility

The inverse compressibility is defined by $\frac{\partial \mu }{\partial n}$. In contrast to transport coefficients such as the conductance $G$, this quantity can be extracted directly from the Landau level spectrum, which makes it a suitable quantity for theoretical considerations. While distinct from conductance, its behavior is related to that of conductance: Divergences in the inverse compressibility indicate positions of filled Landau levels where the conductance exhibits a plateau.

By fixing the temperature $T$, it is straightforward to calculate the charge carrier density $n$ as a function of $\mu$ by populating the energy levels $\left\{{\epsilon }_{i}\right\}$ according to the Fermi function. Each level comes with a degeneracy of $g=\frac{{ABe}}{2\pi \hslash }$, where *A* is system area, leading to a charge carrier density of $n(\mu )=2{\sum }_{i}\frac{Be}{2\pi \hslash }\frac{1}{1+ \exp (\frac{{{\epsilon }}_{i}-\mu }{{k}_{B}T})}$ with the prefactor of 2 accounts for spin degeneracy. The chemical potential $\mu \left(n\right)$ and the inverse compressibility were obtained by a numerical inversion of this function. To this end, we defined a reservoir $M$ of 10^5^ equally spaced values of $\mu$ in a range that was roughly adjusted to the lowest and highest Landau level energies accessible to the considered charge carrier densities. For these values of $\mu$, the carrier densities were computed. The contribution of the lower half of the spectrum (the hole Landau levels) had to be subtracted as an offset.

Fixing the carrier density to ${n}^{*}$, the corresponding chemical potential could then be determined as $\mu \left({n}^{*}\right)=\min \left(\mu \in {M|n}\left(\mu \right) \, > \, {n}^{*}\right)$. The inverse function defined in this way may attain all values from the reservoir from the bottom to the top when $n$ is increased. The numerical error of this procedure is controlled by the spacing within the reservoir. For the practical implementation the temperature entering in the Fermi distribution was chosen to be 0.1 K. This is higher than the usual cryogenic temperatures of the actual experimental realization. However, the resulting broadening may also mimic the finite width of Landau levels due to disorder in the sample.

### Device fabrication

We performed quantum Hall measurements in two different devices. The results from the first device, denoted as Stack 99 in ref. ^47^, are presented in the main text. The following section has been adopted from ref. ^47^

To observe low-energy band structure effects in Bernal bilayer graphene, it is crucial to have high-quality devices. Mechanical exfoliation was used to obtain bilayer graphene, hBN and graphite flakes that were then combined to a delicate 2D heterostructure consisting of a bilayer graphene flake that is encapsulated in hBN, serving as a dielectric material and graphite, serving as electrical gates. Two graphite flakes lie on top of the bilayer graphene and serve as electrical contacts. After exfoliation, the components for bilayer graphene heterostructures were identified and selected using optical microscopy, Raman spectroscopy and atomic force microscopy. Suitable flakes were chosen regarding to their size, cleanliness, and homogeneity. It was ensured that all flakes were free of dirt and wrinkles and that they had not been in contact with chemicals to ensure that they were not contaminated. Thickness measurements via atomic force microscopy revealed the lower hBN flake to be 42-nm thick and the upper hBN flake to be 34-nm thick. A stamping technique^48^ was employed to successively pick up the selected flakes using a home-made “stamping setup^47^” within an argon-filled glove box. A stamp consisting of a block of polydimethylsiloxane (PDMS) used as a cushion layer, and a thin film of polycarbonate (PC) used as a transfer medium, was employed to pick up the individual flakes. A schematic of the stamping process is shown in Supplementary Fig. 1. A detailed step-by-step description of the stamping process is given in the caption of Supplementary Fig. 2 and is based on optical images taken during the process of assembling the device.

Before measuring the electronic properties of the 2D heterostructures, electrical contacts were applied to the conducting layers. Contact lines and pads were added to the graphite contacts and gates using electron-beam lithography, metal evaporation, and wire bonding, enabling electrical connections from the sample to a chip carrier.

The second device (refer to Section E) features four graphite contacts instead of two that go across the bilayer graphene flake as shown in Supplementary Fig. 9a. Since stamping a device with four graphite contacts is more complex and requires additional stamping steps compared to stamping a device with two graphite contacts, the bottom part, consisting of the lower hBN flake and the bottom graphite flake, was stamped first and was then melted onto a clean wafer. Using a new stamp, the upper hBN flake, four graphite flakes serving as contacts and the bilayer graphene flake were then successively picked up. Two of the graphite flakes serving as contacts were located next to each other on the same wafer so that the graphite contacts could be picked up within three steps. The stamp was then melted onto the previously cleaned bottom part. The graphite top gate was stamped in a last step. The thicknesses of the hBN flakes were determined as 58 nm (bottom hBN flake) and 15 nm (top hBN flake) via atomic force microscopy.

### Device characterization

In our dually gated bilayer graphene samples the charge carrier density *n* as well as the electric displacement field *D* can be tuned individually via the use of graphite top and bottom gates. They are defined as

19 $$n={\varepsilon }_{0}{\varepsilon }_{{{{{{\rm{hBN}}}}}}}({V}_{{{{{{\rm{t}}}}}}}/{d}_{{{{{{\rm{t}}}}}}}+{V}_{{{{{{\rm{b}}}}}}}/{d}_{{{{{{\rm{b}}}}}}})/{{{{{\rm{e}}}}}}$$

and

20 $$D={\varepsilon }_{{{{{{\rm{hBN}}}}}}}({V}_{t}/{d}_{t}-{V}_{{{{{{\rm{b}}}}}}}/{d}_{{{{{{\rm{b}}}}}}})/2$$

where *V*_*t*_ (*V*_*b*_) is the gate voltage applied to the top (bottom) gate, *d*_*t*_ (*d*_*b*_) the thickness of the upper (lower) hBN flake serving as a dielectric, *e* the charge of an electron, ${\varepsilon }_{{hBN}}$ the dielectric constant of hBN and ${\varepsilon }_{0}$ the vacuum permittivity.

In order to determine ${\varepsilon }_{{hBN}}$ and to thereby assign *n* and *D*, integer quantum Hall plateaus at finite magnetic fields were aligned with their corresponding slopes in the fan diagram. All observed LL crossings show excellent agreement with those observed previously (see ref. ^29^. where data from the same device is shown). For example, at *B* = 0.4 T (Fig. 4b) one can see the known LL crossings of the *ν* = ± 1 and *ν* = 0 quantum Hall states at *D* ≈ 15 mV/nm as well as crossings at *ν* = ± 2 and *D* ≈ 0 mV/nm.

Having aligned the sample by using the slopes of the quantum Hall states would in principle allow to determine the contact resistance by comparing the measured resistance with the expected quantum Hall resistance. However, due to the use of graphite contacts in a two-terminal device configuration the contact resistance increases linearly with increasing magnetic field (see, for example, Supplementary Fig. 7, more details are given in ref. ^29^). Furthermore, there is a line of decreased conductance across zero displacement field (Supplementary Fig. 6). This region is only bottom- but not top-gate dependent and stems from the region of the BLG that is located below the graphite contacts where the top graphite contacts screen the field of the top gate but not of the bottom gate. Thus, the contact resistance is additionally dependent on the top gate voltage (therefore also on *n* and *D*). To not confuse the reader with the line of decreased conductance we only show the derivative of the conductance in the main text. Another advantage of showing the derivative of the conductance is that it allows to track quantum Hall states at lower magnetic fields where the conductance is not fully quantized yet as traceable fluctuations near incompressible quantum Hall states can appear^27,49,50^. Exemplary, the conductance including a subtracted contact resistance is shown in Supplementary Fig. 7 for *D* = 0 mV/nm and in Supplementary Fig. 8 for *D* = 50 mV/nm. Here a contact resistance was subtracted that linearly increases with *B*. However, we did not account for the dependence on the charge carrier density. Therefore, the resistance values are only valid in a small density regime (negative densities close to the band edge). In Supplementary Fig. 7, we included data taken at larger magnetic fields up to *B* = 1.5 T which we did not show in the main text. At *B* > 0.6 T and *D* = 0 V/nm the quantum Hall states are fully polarized due to additional valley imbalances implying a small residual displacement field. In agreement with previous studies^15,27,41,42^, the even integer quantum Hall states still show wider plateaus compared to the odd integer quantum Hall states.

### Measurements conducted in a second device

Electrical measurements conducted in a second device are shown in Supplementary Fig. 9. The measurements show agreement with the theoretical simulations and the electrical measurements discussed in the main text.

### Supplementary information

Supplementary Information

Peer Review File

## Data Availability

Relevant data supporting the key findings of this study are available within the article and the Supplementary Information file. All raw data generated during the current study are available from the corresponding authors upon request.
