# Supplementary material for: Probing the tunable multi-cone band structure in Bernal bilayer graphene
Source: Nat Commun. 2024 Apr 11;15:3133. doi: 10.1038/s41467-024-47342-0 (PMC11009389; doi:10.1038/s41467-024-47342-0)
Supplement: Supplementary file 1 — Supplementary Information [file 41467_2024_47342_MOESM1_ESM.pdf]

Supplementary Information

for

"Probing the tunable multi-cone band structure in Bernal bilayer graphene"

Anna M. Seiler[1], Nils Jacobsen[1], Martin Statz[1], Noelia Fernandez[1], Francesca Falorsi[1], Kenji Watanabe[2], Takashi Taniguchi[3], Zhiyu Dong[4], Leonid S. Levitov[4], R. Thomas Weitz[1,5]*

[1] 1st Physical Institute, Faculty of Physics, University of Göttingen, Friedrich-Hund-Platz 1, Göttingen 37077, Germany

[2] Research Center for Electronic and Optical Materials, National Institute for Materials Science, 1-1 Namiki, Tsukuba 305-0044, Japan

[3] Research Center for Materials Nanoarchitectonics, National Institute for Materials Science, 1-1 Namiki, Tsukuba 305-0044, Japan

[4] Department of Physics, Massachusetts Institute of Technology, Cambridge, Massachusetts 02139, USA

[5] International Center for Advanced Studies of Energy Conversion (ICASEC), University of Göttingen, Göttingen, Germany

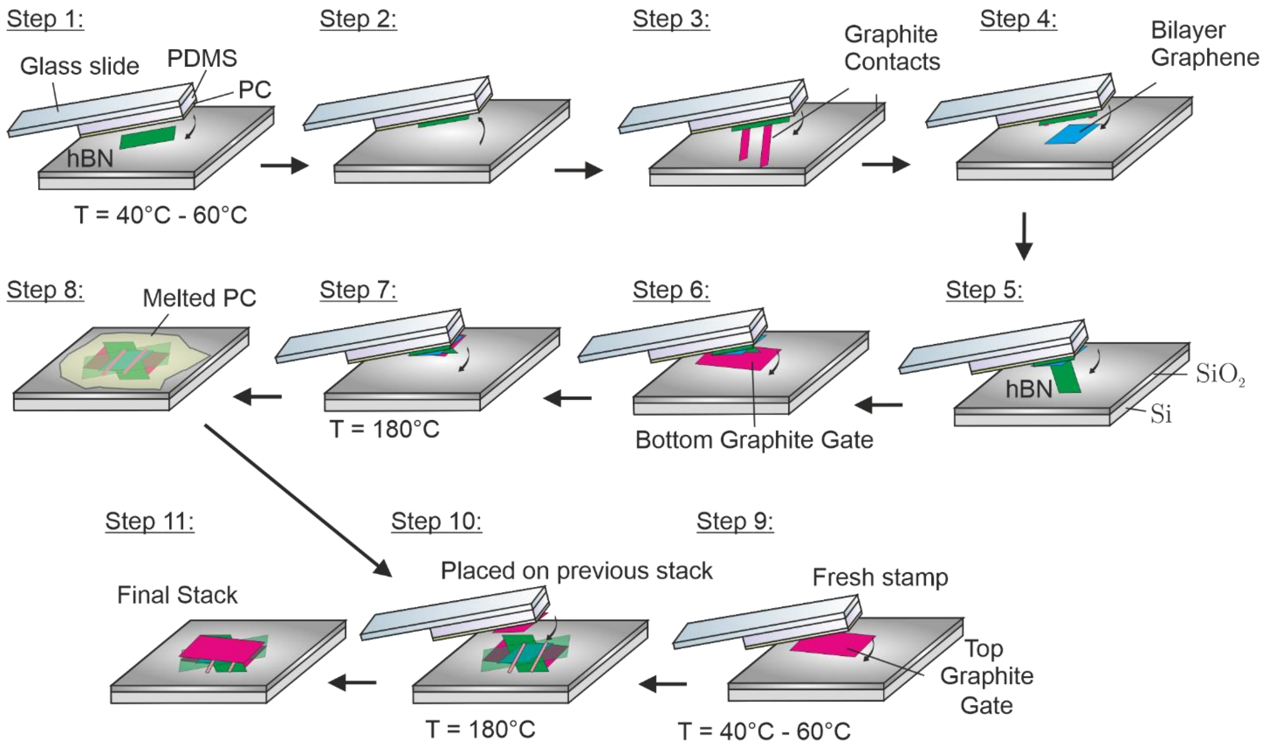

Supplementary Figure 1. Stamping process. Schematic illustration of the stamping process. The graphite contacts can either be picked up together from the same wafer as shown here or from different wafers. Adopted from Ref. [1].

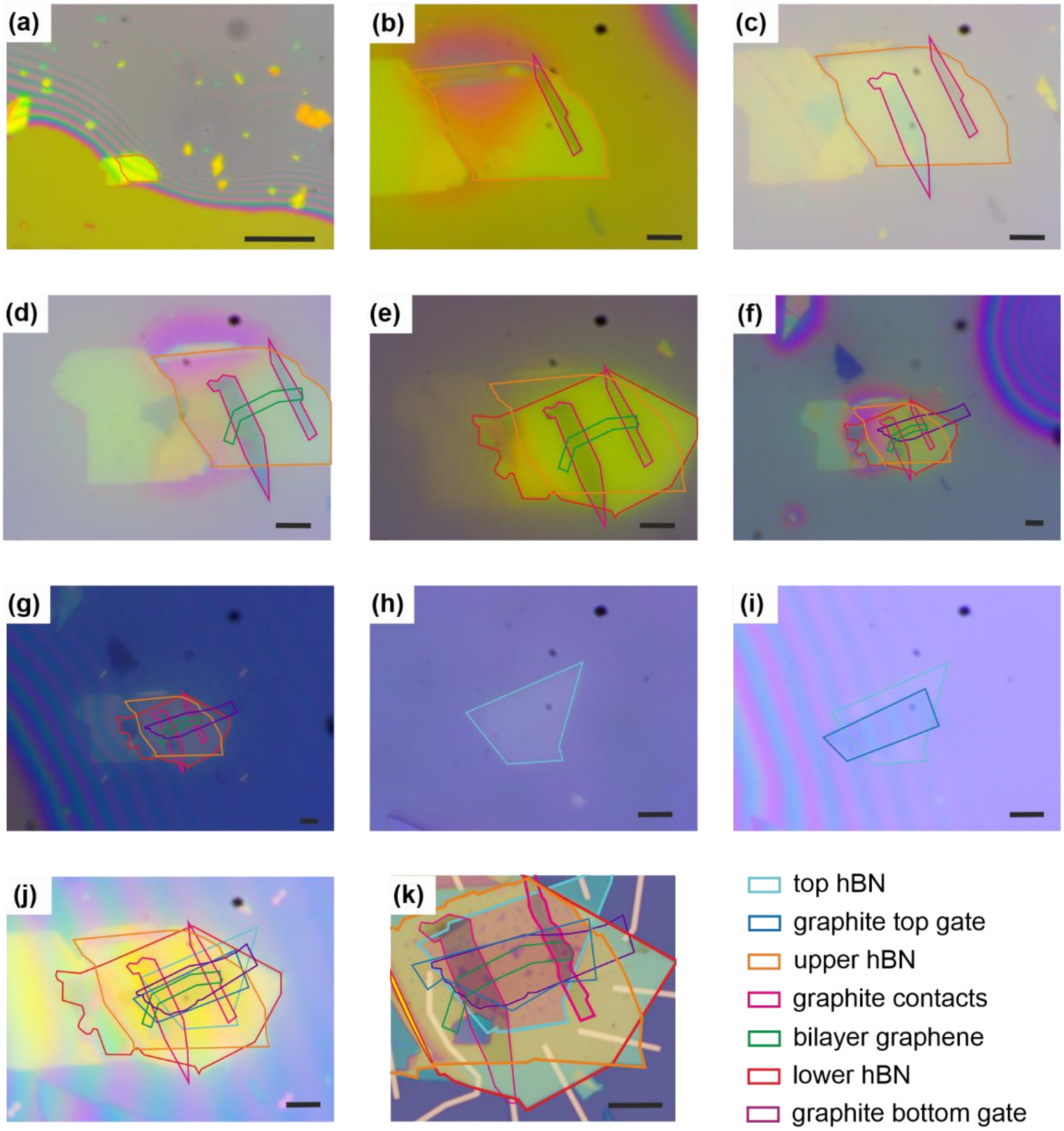

Supplementary Figure 2. **Step-by-step assembling of a bilayer graphene heterostructure.** Sequential optical microscopy images taken during the process of assembling the stack discussed in the main text. The scalebar is 100 µm in **(a)** and 10 µm in **(b)** - **(k)**. **(a)** The stamp is approaching an hBN flake that is outlined in orange and is located on a wafer. It is in contact with the wafer in the lower left part of the image (yellow background) but not yet in contact with the rest of the wafer (grey background). Interference fringes appear in between these two regions. Half of the hBN flake is in contact with the stamp, the other half of the hBN flake is not yet in contact. **(b)** The hBN flake on the stamp is approaching one of the graphite contacts (outlined in pink). **(c)** The upper hBN flake and one graphite contact are located on the stamp, the other graphite contact is located on a wafer. The stamp is approaching the wafer. **(d)** The upper hBN flake and both graphite contacts have been picked up and are located on the stamp. The stamp is in contact with a new wafer that has a bilayer graphene flake on top (outlined in green). **(e)** The bilayer graphene flake was picked up and is now located on the stamp (below the upper

hBN flake and the two graphite contacts). The stamp is approaching the lower hBN flake that is outlined in red. **(f)** The lower hBN flake was picked up, too and the stamp is now in contact with the graphite bottom gate (outlined in purple). **(g)** After picking up the graphite bottom gate, the stack is melted onto an empty wafer that exhibits markers and is heated to 180 °C. The stack as well as the PC film are melted onto the wafer. **(h)** To make it easier to pick up the graphite top gate, a top hBN (outlined in turquoise) is picked up using a fresh stamp. **(i)** The top hBN is used to pick up the graphite top gate that is outlined in blue. **(j)** The top hBN flake and the graphite top gate are both stamped on top of the previously cleaned stack. **(h)** Optical microscope image of the finished stack after it had been further contacted with gold contacts. All flakes are outlined. Adopted from Ref. [1].

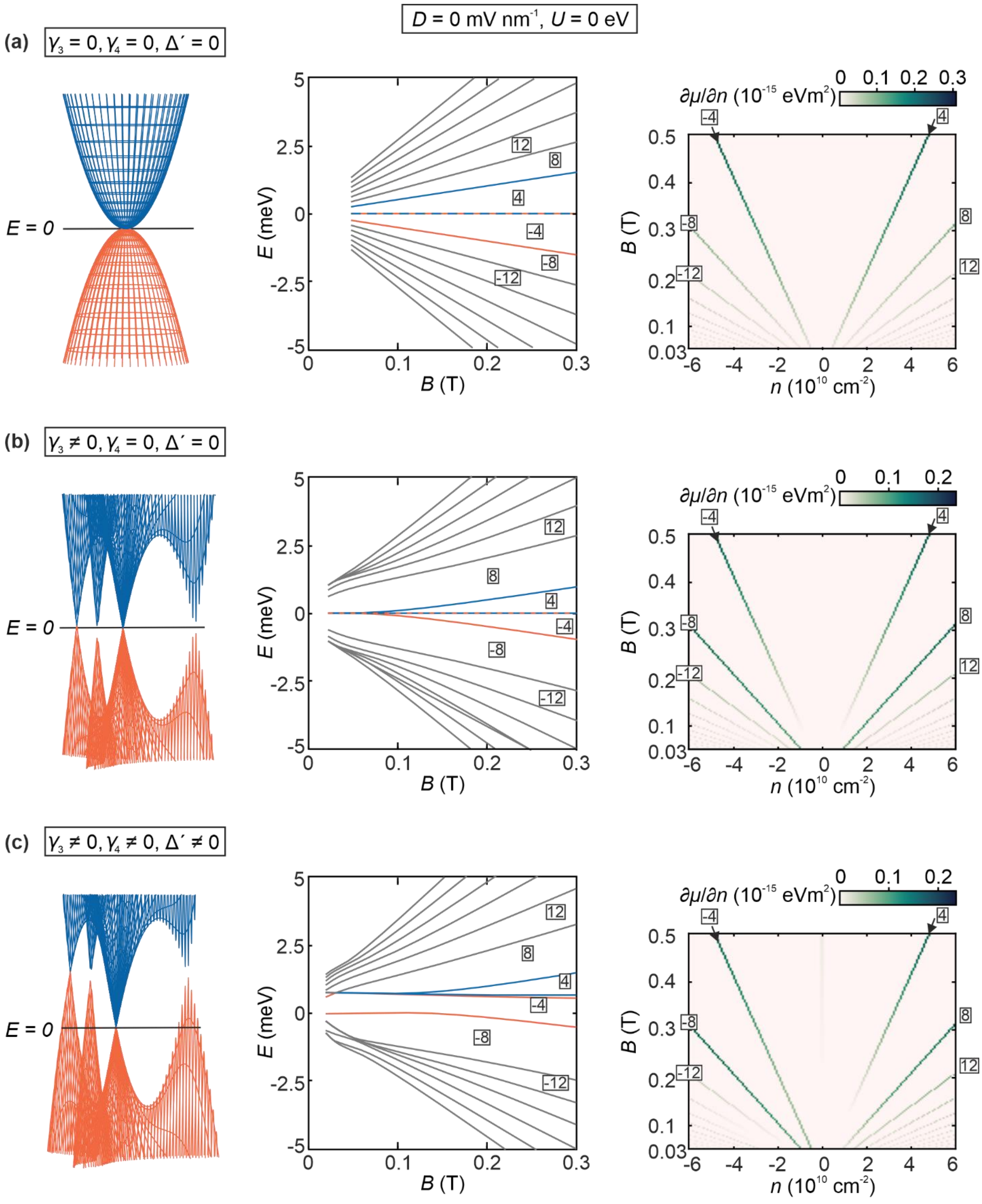

D = 0 mV nm-1, U = 0 eV
(a) γ3 = 0, γ4 = 0, Δ′ = 0
(b) γ3 ≠ 0, γ4 = 0, Δ′ = 0
(c) γ3 ≠ 0, γ4 ≠ 0, Δ′ ≠ 0
E = 0
E (meV)
B (T)
∂μ/∂n (10-15 eVm2)
n (1010 cm-2)

Supplementary Figure 3. Impact of hopping parameters γ_3, γ_4, and on-site parameter Δ' on the band structure, on Landau levels and on quantum Hall states at interlayer potential *U* = 0. The bandstructure is shown in an energy range from -2 meV to + 2 meV (left panel), the evolution of Landau levels as a function of the magnetic field *B* (middle panel), and the calculated inverse compressibility as a function of charge carrier density *n* and magnetic field (right panel) at *U* = 0. **(a)** γ_3, γ_4, and $\Delta'$are not included into the calculations. The parabolic band structure results in four-fold degenerate Landau level and an eight-fold degenerate lowest Landau level. This results in the appearance of quantum Hall states with filling factors *v* = -12, -8, -4, +4, +8, +12, … at very low magnetic fields. **(b)** γ_3 is included into the calculations, γ_4, and $\Delta'$are not included. At low energies, the band structure consists of four mini Dirac cones resulting in a 16-fold degenerate lowest Landau level and in the appearance of quantum Hall states with *v* = -12, -8, +8, +12, … at very low magnetic fields. Quantum Hall states with *v* = -4, +4 appear at *B* > 0.1 T after the magnetic breakdown has occurred. **(c)** γ_3, γ_4, and $\Delta'$are all included into the calculations. This case is discussed in detail in the main text.

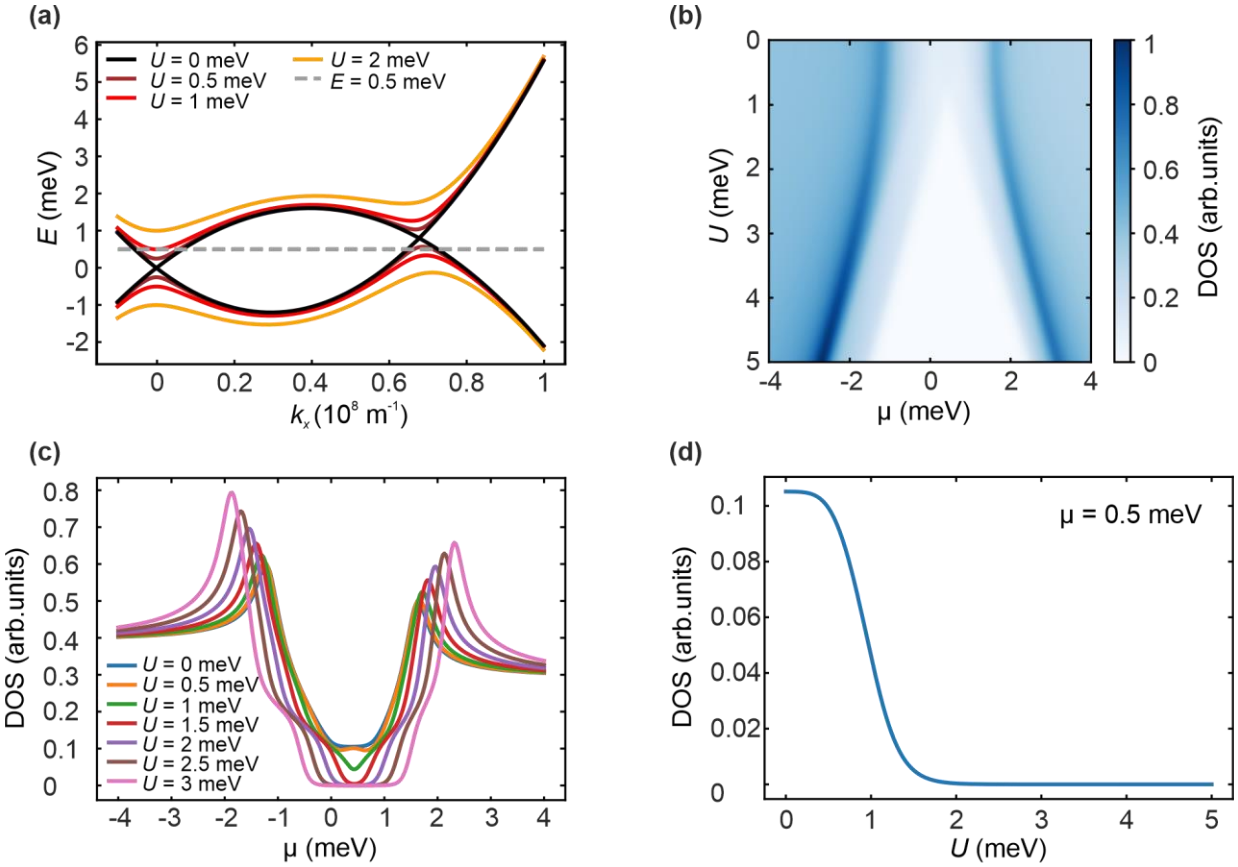

Supplementary Figure 4. Tight binding calculations revealing a semi-metallic regime near the band edge at small *U*. (a) Energy bands as a function of wavevector k_x. For interlayer potential U = 0.5 meV there is no overall gap in the spectrum even though the single pockets are gapped. **(b)** Density of states (DOS) as a function of chemical potential μ and U at temperature T = 1 K. The DOS is approximately constant around μ = 0.5 meV for U < 0.5 meV **(c, d)** Linecuts of the DOS at constant U **(c)** and constant μ **(d)**, respectively.

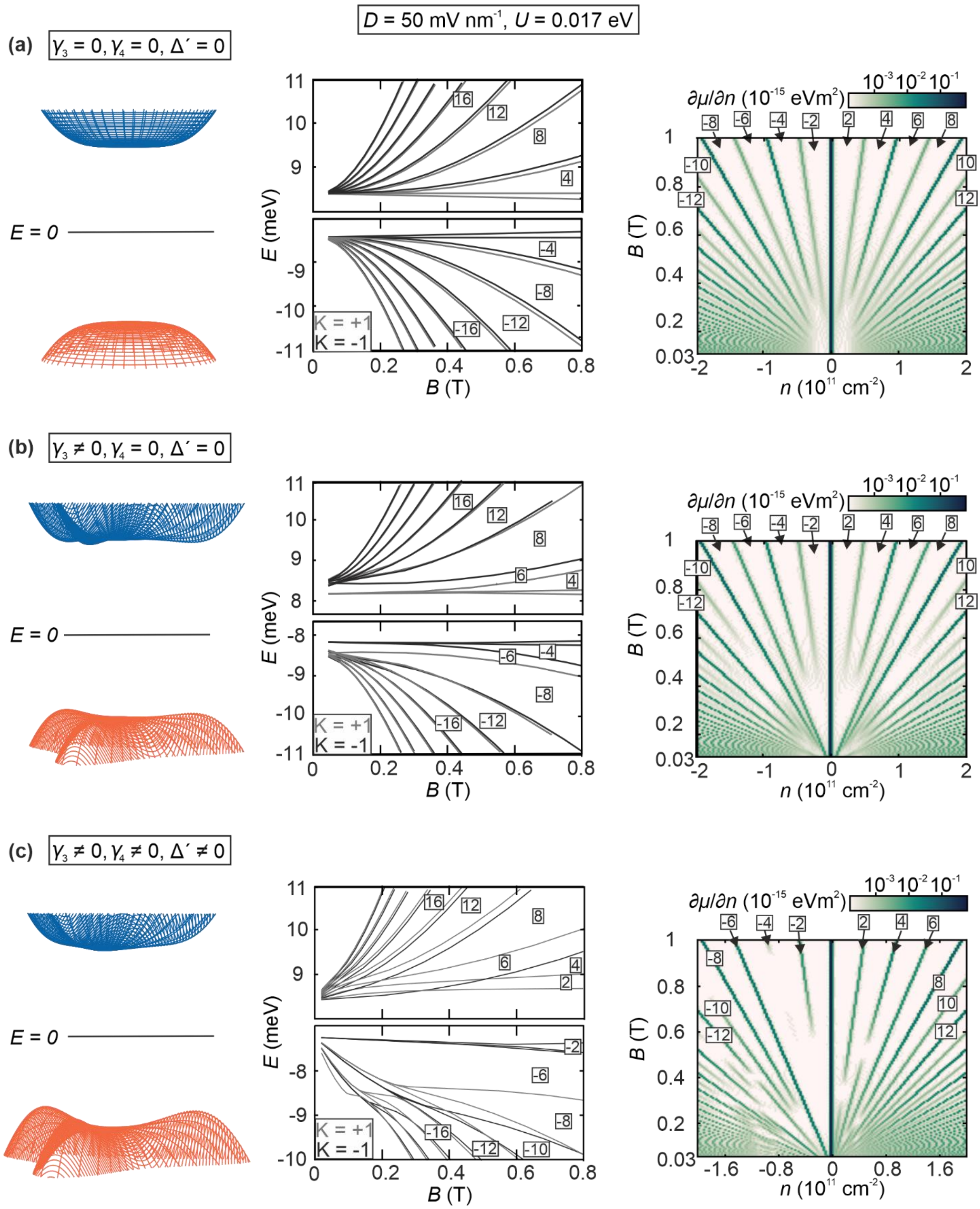

D = 50 mV nm⁻¹, U = 0.017 eV
(a) γ3 = 0, γ4 = 0, Δ´ = 0
(b) γ3 ≠ 0, γ4 = 0, Δ´ = 0
(c) γ3 ≠ 0, γ4 ≠ 0, Δ´ ≠ 0
E = 0
E (meV)
B (T)
K = +1
K = -1
∂μ/∂n (10⁻¹⁵ eVm²)
10⁻³ 10⁻² 10⁻¹
n (10¹¹ cm⁻²)

Supplementary Figure 5. Impact of hopping parameters γ_3, γ_4, and on-site parameter Δ' on the band structure, on Landau levels and on quantum Hall states at interlayer potential *U* = 0.017 eV. The band structure is shown in an energy range from -12 meV to + 12 meV (left panel), the evolution of Landau levels as a function of the magnetic field *B* (middle panel), and the calculated inverse compressibility as a function of charge carrier density *n* and magnetic field (right panel) at *U* = 0.017 eV. **(a)** γ_3, γ_4, and Δ' are not included into the calculations. The almost parabolic band structure results in four-fold degenerate Landau level that split up into two-fold degenerate Landau level with increasing magnetic field. This results in the appearance of quantum Hall states with ν = -4, -2, 0, +2, +4, …. **(b)** γ_3 is included into the calculations, γ_4, and Δ' are not included. At low energies, the band structure consists of three pockets resulting in a 12-fold degenerate lowest Landau level at low magnetic field and in the appearance of quantum Hall states with ν = -6 and +6 at very low magnetic fields. Quantum Hall states with ν = -4, -2, 0, +2, +4, … appear at B > 0.1 T after the magnetic breakdown has occurred. **(c)** γ_3, γ_4, and Δ' are all included into the calculations. This case is discussed in detail in the main text.

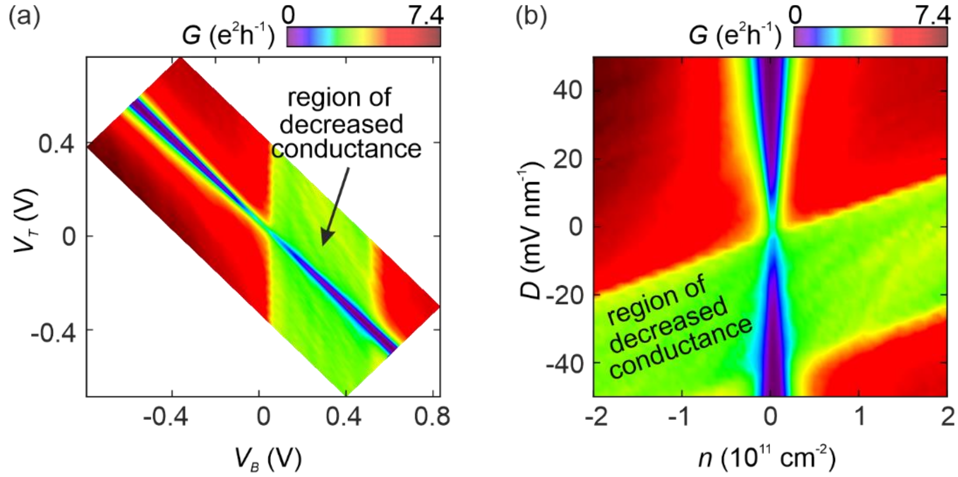

Supplementary Figure 6. Region of decreased conductance. (a) Measured conductance *G* as a function of bottom and top gate in units of e^2h^{-1} with electron charge e and Planck constant h. There is a region of decreased conductance that only depends on V_B but not on V_T. There is no contact resistance subtracted. **(b)** *G* as a function of density *n* and electric displacement field *D* in the same regime that is shown in **(a)**.

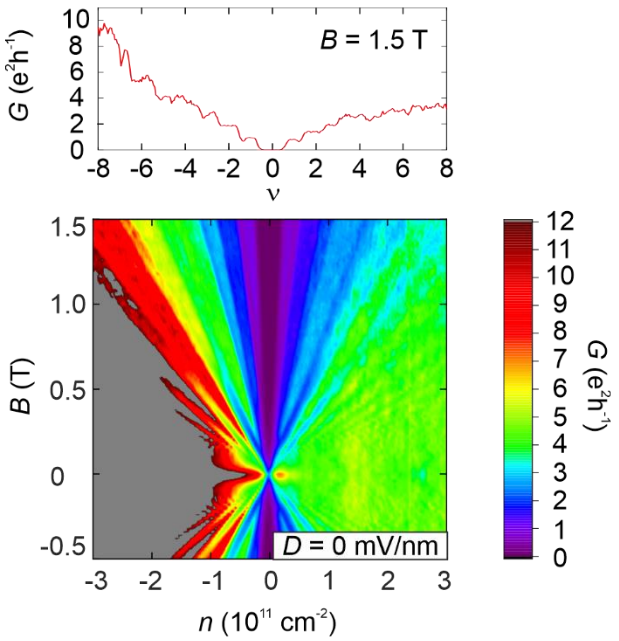

Supplementary Figure 7. Determining the actual conductance as a function of the magnetic field. Conductance *G* as a function of charge carrier density *n* and magnetic field *B* in the same *n*- and *B*- space at electric displacement field $D = 0$ mV nm^{-1} as shown in Fig. 2e. A contact resistance of R_C = 3300 Ω - 4600 Ω T · B^{-1} was subtracted from the measured resistance. Using this value of R_C the quantum Hall states with filling factor $v < 0$ show a quantized conductance where $G = |v|\ e^2h^{-1}$, with electron charge e and Planck constant h, while the conductance values for $v > 0$ are lower due to a higher contact resistance (see Supplementary Figure 6). A line-cut at B = 1.5 T is shown in the top. Here the density *n* is converted into filling factor *v*.

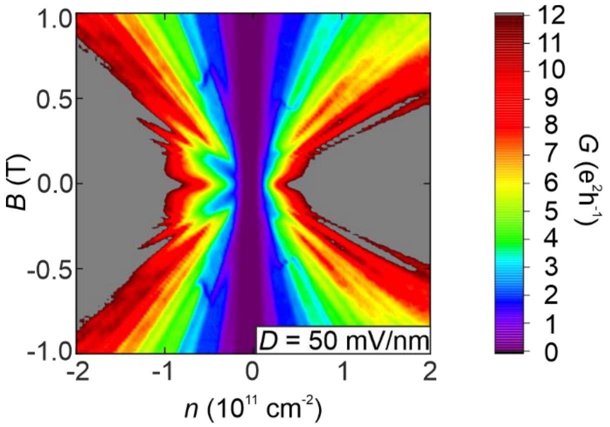

Supplementary Figure 8. Conductance as a function of the magnetic field at a finite electric displacement field. Conductance G as a function of charge carrier density n and magnetic field B at D = 50mV/nm in the same n- and B- space as shown in Fig. 3e. A contact resistance of R_C = 3000 Ω - 3500 Ω T / B was subtracted from the measured resistance. Using this value of R_C the quantum Hall states with $\nu < 0$ show a quantized conductance where $G = |\nu|\, e^2 / h$, with electron charge e and Planck constant h, while the conductance values for $\nu > 0$ are lower due to a higher contact resistance (see Supplementary Figure 6).

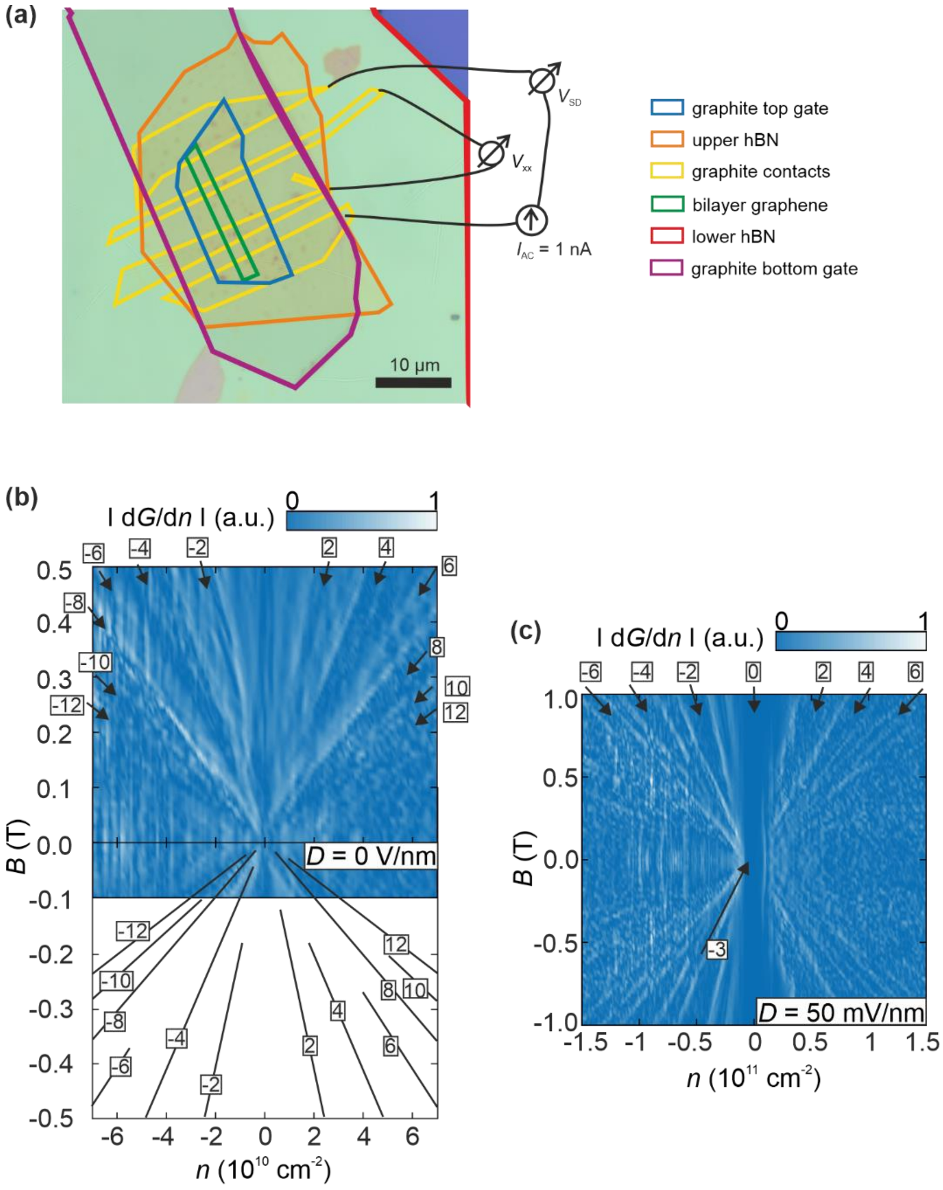

Supplementary Figure 9. Electrical measurements conducted in a second device. (a) Optical microscope image of the second device and corresponding configuration of transport measurements. The different flakes are outlined. **(b, c)** Normalized derivative of the conductance as a function of charge carrier density *n* and magnetic field *B* for electric displacement fields *D* = 0 V/nm **(b)** and *D* = 50 mV/nm **(c)**. Quantum Hall states with *v* = ±8 and *v* = -4 are the most prominent at *D* = 0 V/nm. At *D* = 50 mV/nm quantum Hall states with *v* = -6 and *v* = -3 are the most prominent for hole doping while the quantum Hall state with *v* = +4 is the most prominent for electron doping.

Supplementary References

1. Seiler, A. M. Correlated phases in the vicinity of tunable van Hove singularities in Bernal bilayer graphene. 10.53846/goediss-10001 (2023).
